# Biochemical, Physiological, and Productive Response of Greenhouse Vegetables to Suboptimal Growth Environment Induced by Insect Nets

**DOI:** 10.3390/biology9120432

**Published:** 2020-11-30

**Authors:** Luigi Formisano, Christophe El-Nakhel, Giandomenico Corrado, Stefania De Pascale, Youssef Rouphael

**Affiliations:** Department of Agricultural Sciences, University of Naples Federico II, 80055 Portici, Italy; luigi.formisano3@unina.it (L.F.); christophe.elnakhel@unina.it (C.E.-N.); giandomenico.corrado@unina.it (G.C.); depascal@unina.it (S.D.P.)

**Keywords:** protected cultivation, insect-proof screen, airflow, heat stress, biochemical and physiological responses, functional quality

## Abstract

**Simple Summary:**

Global warming jeopardizes agriculture, which must satisfy the demands of the world’s expanding population for both staple and high-quality products while ensuring increased sustainability. Environmental and regulatory pressure has prompted farmers to convert their production strategies towards sustainable agriculture systems, by introducing for instance, integrated pest management strategies. Insect nets are a suitable tool for pest control but require careful assessment of their effects on the generated microclimate. The low porosity, mandatory for proper exclusion, results in suboptimal airflow and in temperature rise with detrimental effects on crop production and quality. The biochemical and morpho-physiological changes induced by high-temperature impact vegetable crop performance and product quality in advanced growing systems, and also represent a challenge for the most impoverished developing countries of the world, which rely on local horticultural products as a key source of dietary diversity.

**Abstract:**

Environmental pressure poses a major challenge to the agricultural sector, which requires the development of cultivation techniques that can effectively reduce the impact of abiotic stress affecting crop yield and quality (e.g., thermal stress, wind, and hail) and of biotic factors, such as insect pests. The increased consumer interest in premium-quality vegetables requires the implementation of sustainable integrated pest management (IPM) strategies towards an ever-increasing insect pressure, also boosted by cultivation under protected structures. In this respect, insect nets represent an excellent, eco-friendly solution. This review aims to provide an integrative investigation of the effects of the insect screens in agriculture. Attention is dedicated to the impact on growth, yield, and quality of vegetables, focusing on the physiological and biochemical mechanisms of response to heat stress induced by insect screens. The performance of insect nets depends on many factors—foremost, on the screen mesh, with finer mesh being more effective as a barrier. However, finer mesh nets impose high-pressure drops and restrict airflow by reducing ventilation, which can result in a detrimental effect on crop growth and yield due to high temperatures. The predicted outcomes are wide ranging, because heat stress can impact (i) plant morpho-physiological attributes; (ii) biochemical and molecular properties through changes in the primary and secondary metabolisms; (iii) enzymatic activity, chloroplast proteins, and photosynthetic and respiratory processes; (iv) flowering and fruit settings; (v) the accumulation of reactive oxygen species (ROSs); and (vi) the biosynthesis of secondary biomolecules endowed with antioxidant capacity.

## 1. Introduction

The concept of quality has radically evolved driven by a “consumer-oriented” revolution. Nowadays, consumers are more and more sensitive about the nutritional aspects of food and demand attracting high-quality products. According to the consumers’ perception, the functional quality is mainly related to the bioactive phytochemical content. The novel quality concept is supported by consumer interest in the health aspects of food and culinary satisfaction [1]. A product with a high sensory profile and nutritional value is safe, appealing, and sustainable. Interestingly, vegetables are highly rich in water and macronutrients, low in protein and lipids, and are an excellent source of vitamins and minerals, conveying significant benefits such as compounds with antioxidant potential (vitamin C, carotenoids, and phenolics) when included in daily diets [2]. Phenols and polyphenols are natural compounds endowed with reinforcing health repercussions. Recent studies revealed that phenolic compounds safeguard cells during early cancer development (skin, lung, stomach, esophagus, duodenum, pancreas, liver, breast, and colon) [3]. They also exert considerable antioxidant activity with beneficial effects on the vascular and nervous systems, thus reducing the impact of dementia and Alzheimer and Parkinson’s diseases [4]. They are also delineated by having antibacterial, hypocholesterolemic, and hypotriglyceridemic activities [5,6]. Nonetheless, the accumulation of antioxidant molecules is affected by preharvest factors such as genotype; cultivation technique; maturation stage; and climate (e.g., heat, drought, and salinity) [7,8]. 

On average, farmers worldwide harvest about 50% of their potential yield (i.e., the yield they would achieve under optimal growth conditions) [9]. Of this loss, abiotic factors induce about 60–70%, while the other 30–40% is due to biotic stress. These are a challenge to the agricultural sector and require the development of cultivation techniques that reduce the impact of environmental factors, like wind, hail, excessive radiation, and especially, insect damage and thermal stress [10]. The climatic conditions in protected environments foster insect development, such as whiteflies, thrips, and aphids, which cause direct crop damage and transmit phytopathogenic organisms (bacteria, viruses, or fungi), jeopardizing vegetable yield and quality, unless adequately managed [11]. Farmers rely widely on synthetic insecticides for insect control, and researchers have developed more efficient and selective insecticides with reduced environmental impacts. Moreover, we have also witnessed a consistent diffusion of biological pest management methods.

On the other hand, the consumer demand for pesticide-free vegetables and the increased insect resistance to pesticides make insect control always challenging. One of the most important tasks for agriculture remains to contain insect attacks by implementing economically and ecologically sustainable integrated pest management (IPM) strategies. From this perspective, physical barriers are an effective and greener method for reducing chemical insecticides in protected environments [12]. Increasing consumer interest in organic foods and the stricter regulation of chemicals have increased the marketability of anti-insect nets for agriculture. Their performance depends on many factors, like screen mesh and small-hole nets being more efficient [13]. However, small-hole nets are characterized by a high-pressure drop [14], resulting in high airflow resistance, decreased ventilation, and a possible detrimental increase in temperature [15]. 

The sessile state of plants forces them to adapt to a range of environmental stresses. The effect of thermal stress depends on plant tolerance and its ability to adapt quickly to suboptimal conditions, duration, and intensity. Genotype- and environment-dependent adaptive mechanisms ensure their ability to survive and produce under extreme conditions [16]. Plants have a complex set of sensors in different cellular compartments to activate their defense mechanisms as response to thermal stress. These sensors regulate responses to tolerance development. Thermal stimulus-induced response activation is enabled by the interaction of cofactors and signaling molecules capable of activating thermal stress-sensitive genes such as phytohormones, nitric oxide (NO), sugars (as signaling molecules), and Ca-dependent protein kinases (CDPKs) and mitogen-activated protein kinases (MAPK/MPKs) [17]. For example, the increase in membrane fluidity is associated with the activation of signaling cascades coupled to an increase in Ca^2+^ influx, with consequent cytoskeletal reorganization leading to osmolytes and antioxidants production in response to thermal stress [18]. 

Although stress-induced responses are usually multifaceted, life-cycle modification, protective morpho-physiological barriers activation (avoidance or acclimation mechanisms), and the molecular response (tolerance mechanisms) are typical plant reactions to heat stress. Common examples of avoidance and acclimation mechanisms include reducing the absorption of solar radiation by changing leaf orientation (paraheliotropism), reducing water loss by controlling stomatal density, reducing leaf size or abscission, and altering membrane phospholipids [16]. Plants exposed to high thermal stress activate their adaptive response by modifying their morpho-physiological, biochemical, and molecular properties [15,18]. Such stress alters photosynthetic and respiratory processes [19,20,21], impairs flowering and fructification [22,23], reduces enzymatic and chloroplastic activity [24,25], and promotes reactive oxygen species (ROSs) accumulation [26]. As illustrated by Hasanuzzaman et al. [16], high temperatures activate the transcription of heat stress-responsive genes, resulting in the synthesis of signaling molecules; osmoprotectants; nonenzymatic antioxidant compounds such as ascorbate (AsA), glutathione (GHS), tocopherol, and carotene; and enzymatic antioxidant compounds such as catalase (CAT), ascorbate peroxidase (APX), superoxide dismutase (SOD), peroxidase (POX), and glutathione reductase (GR).

Research demonstrated the effectiveness of fine-meshed screens in excluding harmful insects, in addition to the detrimental reduction in airflow due to their use. To date, the main aim of research was to increase airflow by enhancing the intrinsic netting characteristics and to improve growth conditions without affecting exclusion efficiency. However, due to the “antioxidant response” to oxidative stress, high temperatures can alter the intrinsic and extrinsic quality of vegetables, both positively and negatively. A recent study showed the effectiveness of insect nets in enhancing the quality of zucchini squash without affecting yield and, at the same time, ensuring early production [27]. To the best of our knowledge, despite relevant available research papers on the improved airflow of insect nets and their high-temperature effects on the production and quality of horticultural crops, the reviewed literature showed a gap of information in this field of research. The few available contributions suggest that further studies are required to relate the suboptimal growth environment of insect nets to the quality of the produced vegetables, regardless of their exclusion efficiency. 

The aim of this review is to investigate and critically analyze the effects of the insect screens from the plant point of view. The following topics are discussed: (i) the technical aspects of insect nets, (ii) the airflow characterization through screened openings, and (iii) the description of the morpho-physiological and biochemical effects of heat stress on plant growth and yield with a view, in particular, to the antioxidant responses to heat-induced oxidative stress. A literature review was conducted, integrating peer-reviewed papers, books, technical journals, and conference proceedings published by 2020, including technical and physical aspects of insect nets and plants’ responses to high-temperature oxidative stress. 

## 2. Technical Aspects of Anti-Insect Nets

The increasing consumer interest in fresh, sustainable, and high-quality year-round horticultural products prompts the implementation of integrated pest management (IPM) strategies. From this perspective, agro-textiles are a valuable tool for pest management, pollinator confinement, and pesticide reduction. Farmers can rely on different types of insect nets that differ in manufacturing (material, texture, porosity, weight, and number of meshes); radiometric (color, shading, and transmissivity); and physical and mechanical properties [28]. To these purposes, farmers’ concerns are mainly about the best nets, raising several questions. What materials and technical features are ideal for successful exclusion? How do insect nets work? What are the drawbacks of nets? 

A plastic net is a fabric obtained by processing plastic fibers by weaving or nonweaving methods [28]. Woven nets are characterized by regular holes in which air flows due to the connection of vertical warp and horizontal weft threads. In contrast, in a nonwoven net, the fabric is produced by a different process such as extrusion or micro-perforation. The weaving process produces most insect nets available on the market; round or flat plastic monofilaments made of high-density polyethylene (HDPE) or polypropylene (PP) are woven on looms. In agreement with the National Greenhouse Manufacturers Association (NGMA), polyamide (nylon) or multifilament nets in steel and brass or polyethylene and acrylic are marketed, but they have several drawbacks compared to HDPE nets [29]. Steel and brass nets are very resistant and durable, but they are expensive and relegated to the industrial and hobby sectors, while polyamide nets are lightweight but mechanically weak. 

Depending on the texture, as discussed by Castellano et al. [28], three types of insect nets are marketed: Italian, English, and Raschel textures. Italian texture (flat woven net) is produced by overlapping weft and warp threads in orthogonal arrangement. The warp threads are spaced to allow the passage of a weft thread between them, which results in a rigid and stable net. However, when the number of threads per cm^2^ is reduced, the net stability decreases, and the fabric frays when cut. The English texture is a revised and improved version of the Italian one. Two pairs of warp threads are twisted and trapped with weft threads avoiding net fraying. English nets are more stable, resistant, and nondeformable. A complex structure characterizes Raschel-textured nets. The warp threads are knotted to create longitudinal chains that twist and incorporate weft threads. Raschel and English textures are valuable solutions for insect-proof screens. Moreover, they are recommended for anti-hail and windproof nets, where higher tension and resistance are required. 

The weft and warp threads form a regular hole pattern, called mesh, which is the square hole formed at the intersection of a warp and weft thread, varying from 0.2 to 3.1 mm, depending on the insect size to be excluded [28]. Insect nets available on the market are described by mesh number, representing the number of holes per inch in each direction [30]. The insect’s exclusion is based on avoiding insect thorax passage (“prison effect”) [31], and, theoretically, a net is efficient when the holes are smaller than the thorax width of the insect to be excluded. This parameter also depends on the insect sex [32]. Table 1 shows the average thorax width of “key insects” and the hole size and mesh number required for their effective exclusion from greenhouses. The hypothetical exclusion efficiency does not necessarily coincide with real effectiveness, achieving up to 90% control of a designated pest [33]; for example, due to the shape of thrips (*F. occidentalis*) bodies, they can penetrate through small holes of widespread commercial nets [34]. The reason that small holes do not ensure total exclusion is correlated to the 3D arrangement of the threads. Usually, nets are considered flat structures, but they are three-dimensional, and their effectiveness depends on several factors like the threads’ thickness, width and length of the hole, and its geometry [34]. Warp threads are usually closer together than weft threads, forming a hole with a rectangular geometric structure; the overlapping of warp and weft threads alters the geometric structure of the hole, allowing easy access of the insect [34].

Manufacturers do not have specific tools to evaluate insect nets’ efficiency. Therefore, several laboratory experiments were carried out to assess the exclusion efficiency of different types of nets in calm conditions and at different air velocities and temperatures [35,36,37]. In recent years, the agro-textile industry has tested and marketed innovative nets with improved airflow, due to thinner threads, without affecting exclusion performance. A recent experiment carried out by Formisano et al. [27] investigated the effects of a suboptimal growth environment induced by two 50-mesh nets with different porosities (Biorete^®^ 50 mesh and Biorete^®^ 50 mesh AirPlus, Arrigoni S.p.A, Uggiate Trevano, CO, Italy) on the production and quality attributes of *Cucurbita pepo* L. in controlled growing conditions. The improved porosity of the 50-mesh AirPlus net, due to a thinner HDPE filament (Harlene HT^®^, Arrigoni S.p.A, Uggiate Trevano, CO, Italy), resulted in increased quality traits of zucchini squash without compromising yield. The 50-mesh AirPlus net led to an improvement in the inner microclimate, with lower soil and air temperatures and relative humidity. A comparable study on cucumber showed the positive effects of insect-proof screens with different porosities in containing cucumber beetles in high tunnels while providing adequate ventilation [38]. 

The durability and mechanical stability of the nets are essential parameters, and fabrics with complex textures confer enhanced mechanical characteristics, increasing the stability. However, durability does not depend exclusively on the number and structure of the threads. Several elements, such as environmental factors (temperature), chemical treatments, dirt, and UV radiation, affect the mechanical and physical characteristics of plastic threads, leading to premature net deterioration. UV radiation plays a crucial role in the lifetime and performance of nets [39]; hence, manufacturers use additives to increase the UV stability of HDPE plastic polymers. The longevity of nets is directly related to their resistance to UV radiation, which is expressed in the amount of kiloLangley (kLy) and represents the number of years required to reduce the net tensile strength by 50%. For example, a net with 600 kLy in a Mediterranean climate region (100–130 kLy) potentially has a lifetime of five to six years [28]. 

Insect nets are usually made with transparent or white fibers; however, the industry has recently tested multifunctional nets supplying protection and photoselection by adding colored and UV-absorbing additives to HDPE polymers. Many authors reported that light modulation using photoselective nets induces a “barrier effect” against pests while reducing the incidence of viral diseases affecting horticultural crops. Antignus et al. [40] reported that UV-absorbing plastic screens were effective in decreasing the dispersion rate of pests in greenhouses. Whiteflies detect solar radiation in a specific light spectrum, and their findings showed that the lack of UV radiation in greenhouse interferes with the flight and orientation of insects. Further studies conducted by Legarrea et al. [41] investigated the impact that UV-absorbing nets had on the visual cues of two beneficial predators (*Orius levigatus* and *Amblyseius swirksii*). The results obtained showed that the lack of UV radiation created a favorable environment for *Orius levigatus*, in contrast to what occurred with *Amblyseius swirksii*. In a comparative study, Ben-Yakir [42] evaluated the impact of colored photoselective nets (yellow, red, and pearl ChromatiNets™, Polysack Plastic Industries, Nir-Yitzhak, Israel) on the containment of aphids and aleyrodids involved in the transmission of the potato virus Y (PVY), cucumber mosaic virus (CMV) in peppers, and the tomato yellow leaf curl virus (TYLC). Specifically, yellow and pearl nets reduced aphid and whitefly infestation up to three-fold compared to red and conventional black nets. Similarly, yellow and pearl nets reduced the incidence of CMV, PVY, and TYLC up to ten-, three-, and four-fold, respectively.

Over the last two decades, various pest management methods were implemented, such as insecticide-treated insect nets. Studies on cucumbers (*Cucumis sativus* L.) and African eggplants (*Solanum macrocarpon* L.) demonstrated the efficacy of pyrethroid-treated nets in the management of aphids and Lepidoptera, although providing lower efficacy in containing tiny insects such as whiteflies (*Trialeurodes vaporarium*) and thrips (*Frankliniella occidentalis*) [43,44]. In a recent trial, Arthurs et al. [45] tested the exclusion performance of a two-colored modern long-lasting insecticide net (LLIN) with a larger mesh size (32 holes/cm^2^) compared to a conventional thrips exclusion screen. The results showed lower thrips penetration in yellow-treated nets than in black ones. However, while insecticide-treated nets resulted in considerable airflow increase, a larger hole size did not guarantee total thrips exclusion.

Insect nets are commonly used in agriculture, and their effectiveness is proved by many studies. Nets represent a valuable eco-sustainable solution to limit the use of pesticides, thus exposing producers and workers to lower risks. The requests of the globalized market have driven technicians, producers, and researchers to consider insect nets as multifunctional tools that provide high exclusion efficiency, environmental and economic eco-sustainability, and that ensure high yields and high-quality products. In previous decades, research has focused on improving airflow to limit the detrimental impact of excessive temperatures in the warm Mediterranean regions. High temperatures, if critical thresholds are not exceeded, can ensure an early production and an improvement in the quality of vegetables, such as a higher antioxidant content. Despite extensive research on the plant response to high temperatures, few studies have examined the possible improvement in quality caused by the insect nets, as well as the most appropriate porosity level, to ensure a balance between the production, quality, and efficiency of exclusion.

## 3. Airflow Characterization of Screened Openings

To ensure optimal growth conditions in protected environments, it is necessary to provide adequate ventilation, especially in warm Mediterranean regions. High solar radiation and insufficient ventilation cause a rapid rise in air temperature, exposing crops to severe stress affecting all growth stages and crop production [16]. For sufficient air exchange, vents should be 15% to 25% of the total area and should cover the entire length of the greenhouse for balanced air distribution [30]. The air flowing through the greenhouse moves according to a pressure gradient. The air exchange process occurs either by natural (passive) or forced ventilation [47], each aimed at replacing warm indoor air with cooler air from the outside. With natural ventilation, the airflow through the vents is triggered by temperature differences and wind pressure, but mainly wind contributes to air renewal [48]. The airflow drives insects through the openings, and, therefore, insect nets are usually mounted on greenhouse openings like doors and vents [30]. The exclusion performance depends on the mesh and hole geometry [13,32]. Fine-meshed nets, despite their theoretical better exclusion efficiency, have the disadvantage of low porosity (percentage of the ratio between open net area and total net area). Consequently, a high-static pressure drop occurs [14], leading to inadequate air exchange and rising temperature and humidity [49]. 

Despite the availability of advanced solutions to increase net porosity without reducing mesh size, thereby improving air exchange in protected environments, it is still necessary to estimate the pressure drop that occurs through screened openings [30]. From a physical perspective, the air is a viscous and compressible fluid with a variable velocity, which moves according to either the laminar or turbulent regime. Viscous forces govern the movements in a laminar flow, while, in a turbulent flow, inertial forces are also involved. Considering air as an incompressible fluid (constant density), the only variable that discriminates from the turbulent and laminar flow is the Reynolds number (*Re*). For insect net, the Reynolds number is defined as follows:

$$R_{e}=\frac{ud}{\nu }$$

where:

$u=\mathrm{flux}\;\mathrm{velocity}\;\left(m/s\right),$
$d=\mathrm{thread}\;\mathrm{diameter}\;\left(m\right),$ and
$\nu =\mathrm{kinematic}\;\mathrm{viscosity}\;\left(m^{2}/s\right).$

It is a dimensionless parameter that physically expresses how the inertial and viscous forces acting on a fluid particle move at *u* velocity. When air flows through a screened opening, the flow rate decreases significantly with the pressure drop that occurs from the inside out. Therefore, a prediction of the total pressure drop through insect-proof screens is necessary to ensure their correct sizing and, consequently, sufficient air exchange without compromising the exclusion efficiency. The total pressure drop ΔP_T_ is the sum of the pressure drop caused by unscreened opening and insect screen [49] and is given by:

$$\Delta P_{T}=\Delta P_{o}+\Delta P_{s}$$

where:

$\Delta P_{o}$ = pressure drop across the unscreened opening, and
$\Delta P_{s}$ = pressure drop across the screen $\left[\mathrm{Pa}\right].$

The pressure drop generated by insect nets can be assessed both through a “coefficient of discharge” included in Bernoulli’s equation [50,51,52] and by the motion equation of a fluid through a porous medium (Forchheimer equation) [53,54]. Supposing that air moves by turbulent flow (*Re* > 150), it is possible to quantify the pressure drop and the airflow through an unscreened opening using Bernoulli’s equation. A fluid movement through an opening is subjected to a contraction, causing in the flow an effect known as vena contracta (*V_c_*), which represents the fluid flow point where the section is minimal, the velocity is uniform, and the static pressure is equal to the surrounding air [55]. The ratio between the vena contracta and the total area of a hole (*A*) defines the contraction coefficient (*C_c_*):

$$C_{c}=\frac{A_{c}}{A}$$

As a result of hole contraction, the velocity in the vena contracta is lower than ideal velocity (*V_i_*); the equation that correlates the two velocities is defined as velocity coefficient (*C_v_*):

$$C_{v}=\frac{V_{C}}{V_{i}}$$

Outside and inside the net, we have, respectively:

$$\frac{\rho }{2}*V_{0}^{2}+P_{0}=\frac{\rho }{2}*V_{i}^{2}+P_{i}$$

where:

$V=\mathrm{fluid}\;\mathrm{velocity}\;\left(m/s\right),$
$P=\mathrm{static}\;\mathrm{pressure}\;\left(\mathrm{Pa}\right),$ and
$\rho =\mathrm{fluid}\;\mathrm{density}\;\left(\mathrm{Kg}/m^{3}\right).$

For the ideal fluid, without friction, the velocity is different from the real one; assuming the external velocity as zero, we obtain the equation that relates the ideal (or theoretical) velocity to the static pressure variation:

$$V_{i}=\sqrt{2*\frac{P_{0\;}-\;P_{i}}{\rho }}$$

The continuity equation, describing the airflow through an opening, can be defined as follows:

$$Q=\;A_{c}*V_{C}=\;C_{c}*A*C_{v\;}*\;V_{i}=C_{c}*A*C_{v\;}*\sqrt{2*\frac{P_{0\;}-\;P_{i}}{\rho }}$$

The multiplication between the contraction coefficient and the velocity coefficient is defined as the discharge coefficient (*C_d_*), expressing the resistance that a specific opening offers to the airflow [48]. 

Therefore:

$$Q=\;C_{d}*A*\sqrt{2*\frac{P_{0\;}-\;P_{i}}{\rho }}$$

Experiments were carried out to determine the discharge coefficients of the openings, as well as the nets. The discharge coefficients of vents ranged from 0.60–0.90 [56,57] as a function of the sharp edge, whereas they ranged from 0.05 to 0.5 as a function of net porosity [58,59]. The flow resistance is often expressed by the pressure loss coefficient (*K*), correlated to the discharge coefficient by the following relationship:

$$K=\frac{1}{C_{d}^{2}}$$

Based on previous observations, the pressure drop through an unscreened opening is given by the equation below:

$$\Delta P_{o}=\frac{1}{2}K\rho V^{2}$$

Moreover, several researchers developed correction functions to adjust the pressure loss value by correlating the pressure loss coefficient to the aspect ratio (L/H) of the openings [60] and considering the influence of flaps [48]. Usually, insect nets have an ideal Reynolds number below 150, which results in a laminar flow [61]; therefore, it is known that the pressure loss coefficient is a function of both the porosity and Reynolds number [62].

In the literature, numerous research have linked the *K* coefficient to different porosity values with different *Re* values [48,63,64]. Net resistance to airflow can be evaluated by the physical laws governing the movement of a fluid through porous media. From this viewpoint, nets are assumed as solid porous structures consisting of interconnected holes. On a small scale, the pressure drop is usually expressed by Forchheimer’s equation:

$$\frac{\partial \;P}{\partial \;x}=\frac{\mu }{K}v+\rho \frac{Y}{K^{1/2}}\left|v\right|v$$

The infinitesimal pressure drop is the sum of a linear term, reflecting the flow resistance generated by the viscosity *μ* and the specific permeability *K* of the porous medium and a quadratic term depending on the permeability of the medium *K* and the inertial factor (*Y*) (relative to the pore characteristics) [53]. Different *K* and *Y* values were reported by Miguel [53] and Valera [54] and were classified based on screen porosity. 

As cited by Succi and Vulpiani [65], the fluid flow in porous media is dominated by a high prevalence of dissipative over convective processes. Therefore, at a low Reynolds number (*Re* < 1), the flow can be described by Darcy’s law (linear term of Forchheimer’s equation); in particular, the nonlinear term can be ignored, and the flow velocity shows a linear trend with pressure loss:

$$\frac{\partial \;P}{\partial \;x}=\frac{\mu }{K}v$$

with a Reynolds’ number over the unit (1 < *Re* < 100), nonlinear effects cannot be ignored [61,65]. 

The applicability of Bernoulli and Forchheimer’s equations is dependent on the Reynolds’ number. At *Re* > 150, the pressure drop can be determined by the discharge coefficient of Bernoulli’s equation, whereas the laminar flow rate (*Re* < 150) by Forchheimer’s equation. Teitel [66] and Kittas et al. [50] demonstrated that the variations in pressure drop obtained with the two mentioned methods were relatively small. On the other hand, at *Re* > 8, the pressure drop can be determined by the discharge coefficient [66], although it is not constant at all values of the Reynolds number, according to Teitel and Shklyar [14].

Insect nets are effective ecological solutions in regulating pests. However, as shown in the published literature, low-porous nets drastically decrease the ventilation rate, resulting in higher relative humidity and temperature gradients in protected environments (Table 2). As mentioned by Ajwang et al. [41], the airflow improvement can be achieved by adequately sizing the screened openings according to the pressure drop produced by the net. A correction factor, relative to net porosity, was proposed by Perez-Parra et al. [67] to improve the ventilation area. However, as suggested by Fatnassi et al. [68], it is not always possible to compensate the pressure drop by increasing the screened area; therefore, a forced ventilation system is required in this case.

## 4. Morphological, Physiological, and Biochemical Responses of Plants under Heat Stress

### 4.1. Effect of Heat Stress on Growth and Yield

It is well-documented that very intense solar radiation and thermal stress negatively affect crop physiology with, for instance, significant yield and quality losses in cereals, legumes, and vegetables [7,18]. High temperatures affect all growth stages, especially germination and reproduction. Common and early effects caused by high temperatures are necrosis; leaf elongation (hyponastia); drying and burning of leaves, branches, twigs, and stems; fruit discoloration and damage; leaf abscission; poor germination and rooting; loss of turgidity; and cell size reduction, leading to a decrease in total biomass [22,75]. The plant can also manifest programmed cell death (PCD), causing leaves, flowers, and fruits to fall and, in extreme cases, the whole plant to die [76]. Germination, mostly the development of the embryo axis and its emergence, is particularly sensitive to temperature fluctuations. Short exposure to high temperatures can lead to a reduction in the percentage of seed germination or a total inhibition, as well as poor vigor and reduced plant, rootlets, and plumules growth [77].

Considerable high temperature effects were recorded in several crops, affecting their quantitative and qualitative characteristics. In Leguminosae such as the common bean (*Phaseolus vulgaris* L.) and peanuts (*Arachis hypogea* L.), high temperatures reduced the yields [78,79]; similarly, in tomatoes (*Lycopersicum esculentum* Mill.), Camejo et al. [80] reported a significant yield reduction due to defects in embryo fertilization and meiosis. In many cultivated species, the effects of heat stress are more evident in reproductive development than in vegetative growth. All plant tissues are susceptible to high temperatures, and a few degrees increase during anthesis can lead to significant yield losses [18]. According to Zinn et al. [81], high temperatures shorten the number of days to anthesis, hampering the optimal nutrients accumulation for embryo development. Further studies on tomatoes, snap beans, and zucchinis showed tapetum degeneration and pollen sterility caused by PCD and endoplasmic reticulum malformations [82,83]. Under heat stress, it is likely that the under-regulation of sucrose synthetase and pollen vacuolar invertases occurs, as verified in tomatoes and cowpeas [84]. A further relevant effect induced by high temperature is the abscission of reproductive organs due to increased levels of abscisic acid (ABA) and ethylene (ET), combined with altered or reduced auxin (AUX) biosynthesis [85].

### 4.2. Plant Physiological Response to Heat Stress

Heat stress affects a range of physiological processes that are essential for the proper functioning of cell structures. High temperatures hamper water and nutrient uptake and impair most physiological and photosynthetic functions, leading to reduced productivity and economic return [86]. The proper functioning of metabolic processes in plant tissues requires adequate tissue hydration. High temperatures, however, lead to a rapid reduction in the water contents in leaf tissue and soil; a decrease in root conductance, as in tomatoes [87], mass, and growth [7]; and a decline of the activity of critical enzymes, such as nitrate reductase [88], essential for nutrient uptake, as well as for source and sink activity [89]. 

Photosynthesis is the most sensitive to heat stress among plant physiological processes. Complex reactions leading to CO_2_ reduction involve thylakoid reactions (specialized internal chloroplastic membranes) and carbon-fixing reactions. Foliar mesophyll cells are rich in chloroplasts, with pigments for light absorption (chlorophylls). In chloroplasts, light energy is captured by two distinct photosystem units (PSI and PSII) and used to trigger electron transfer to reduce NADP^+^ and oxidize H_2_O. Therefore, under heat stress, an optimal performance of cell membranes might support a better photosynthetic and respiratory efficiency. However, high temperatures have shown to affect cell structures negatively and, thus, photosynthesis as well. Specifically, they alter the structure of chloroplasts [25], reduce the enzymatic activity of ribulose 1,5-biphosphate carboxylase (RuBisCo) and its regeneration, as shown in cotton plants [90] and RuBisCo activase [87,91], induce the closure of stomata by decreasing the CO_2_ availability and, consequently, the activity of RuBisCo [92], which is recognized to have a low affinity toward CO_2_ compared to O_2_ [93], reduce carbon fixation with oxygen evolution, and generate reactive oxygen species (ROS) [80,94]. Notably, damage to photosynthetic pigments was observed, probably due to lipid peroxidation of chloroplasts and thylakoids, the reduction or stop of PSII activity, and reduction of electron flux and maximum PSII quantum efficiency (Fv/Fm ratio) [20,21]. Chlorophyll’s lower accumulation is due to its reduced biosynthesis, degradation, or effects of either due to the deactivation of crucial enzymes such as 5-aminolevulinate dehydratase, as studied in cucumbers [95,96]. Camejo et al. [80] also observed an increase in the chlorophyll a/b ratio and a decrease in the chlorophyll/carotenoid ratio of heat-tolerant tomato cultivars.

### 4.3. Biochemical Response to Heat Stress: The Role of Antioxidant Compounds

In response to heat stress, plants maintain their physiological function through self-regulating mechanisms (i.e., homeostasis) by producing and accumulating a wide variety of osmoprotectants (i.e., “compatible solutes”) to restore osmotic pressure [97]. Plant cells have numerous compounds, like proline, glycin-betaine, betaine, soluble sugars, sugar alcohols or tertiary and quaternary ammonium compounds, ubiquitin, dehydrins, and late-embryogenesis-abundant (LEA) proteins [7,98]. These compounds also prevent the deactivation of critical enzymes such as RuBisCo under high temperatures, scavenging free radicals and stabilizing subcellular structures [20,99,100,101]. In addition to compatible solutes, several authors also agree that soluble sugars, such as glucose and sucrose, play a direct role in heat stress tolerance by regulating carbon allocation, acting as signal molecules [102,103], protecting pollen cells by enhancing their quality, as in tomatoes [104], and acting as antioxidants and ROS scavengers at high concentrations [105,106]. 

Thermal stress produces harmful reactive oxygen species (ROS, e.g., compounds with high oxidizing activity and a strong tendency to donate oxygen atoms to other substances) [7], triggering a “chain” reaction that can be stopped by antioxidant compounds. ROS can be divided into two main categories: free radicals, such as hydroxyl radical (OH^•^), nitroxide radical (NO^•^), superoxide anion (O_2_^•−^), and singlet oxygen (O^•^) and nonradical species, such as hydrogen peroxide (H_2_O_2_) and ozone (O_3_) [107]. ROS production occurs mainly in chloroplast reaction centers, peroxisomes, and especially, in the mitochondria by enzymatic and nonenzymatic pathways [107], by photo-oxidation reactions, Haber-Weiss and Fenton reactions, mitochondrial electron transport chain reactions, and during photo-inhibition [108,109]. The superoxide radical anion (O_2_^•−^) does not possess high reactivity. It is not able to pass through the mitochondrial membrane, and its formation occurs spontaneously during cellular respiration by cytochrome oxidase that releases partially reduced intermediate compounds, including O_2_^•−^ and H_2_O_2_. 

Even though H_2_O_2_ is not a radical species and does not cause any immediate risk to cell structures, it is involved in the synthesis of reactive ROS. Its formation can also occur due to the enzyme superoxide dismutase (SOD) from two molecules of superoxide anion. The hydroxyl radical (OH^•^) production, which has a high reactivity towards biomolecules, causing considerable cellular damage, is based on H_2_O_2_ and O_2_^•−^ use in Haber-Weiss and Fenton reactions:

$$O_{2}^{•-\;}+H_{2}O_{2}\to {\;\mathrm{OH}}^{•}+{\mathrm{OH}}^{-\;}+O_{2}\;\left(\mathrm{Haber}-\mathrm{Weiss}\;\mathrm{reaction}\right)$$

$${\mathrm{Fe}}^{2+}+H_{2}O_{2}\to {\;\mathrm{OH}}^{•}+{\mathrm{OH}}^{-\;}+{\;\mathrm{Fe}}^{3+}\;\left(\mathrm{Fenton}\;\mathrm{reaction}\right)$$

Overexposure to ROS causes oxidative stress that leads to the activation of many cellular antioxidant systems. These are activated to avoid any damage to proteins, enzymes, lipids, photosynthetic pigments, and other cellular components. Oxidative damage results in protein denaturation and membrane instability; lipid peroxidation; photosynthetic reaction center damage; thylakoid membrane electron leakage; impairment; reduced biosynthesis; and reduced accumulation of metabolites, carbohydrates, enzymatic activity, and osmotic imbalance [26]. Oxidative stress is, therefore, the natural expression of a damage that occurs when pro-oxidant factors (abiotic and biotic pressures) exceed the endogenous antioxidant defenses.

One of the most frequent oxidative alterations occurs in lipids, causing a “chain mechanism” (lipoperoxidation) in the polyunsaturated fatty acids of membrane phospholipids. The reaction chain produces reactive compounds such as malondialdehyde (MDA), able to react with free amino groups of proteins, phospholipids, and nucleic acids, inducing molecular structural alterations [110]. The reaction ends when no more oxygen is available or by the action of antioxidants that donate an atom of hydrogen or an electron, forming nonradical inactive species. However, ROS also acts as a molecular signal, enabling complex metabolic reactions by which the plant activates thermal stress defenses. Mittler et al. [111] highlighted the vital role of ROS in promoting transcription and translation processes in chloroplasts, necessary to develop defenses against high temperature-induced oxidative stress. Environmental stresses prompt ROS production in plants that react by modulating their antioxidant metabolism [76]. Plants undergo high oxidative stress due to harmful ROS under thermal stress and synthesize a wide range of antioxidants, which lead to an increased stress tolerance. The ROS removal is necessary for cell survival, and several studies have shown that antioxidant compounds of enzyme and non-enzyme origin are widely produced in all cell structures under stress conditions [107,111].

Effective plant defense chemicals are nonenzymic, low-weight antioxidant compounds (i.e., “scavengers”), such as glutathione (GHS), ascorbic acid (AsA), α-tocopherol, phenolics, carotenoids, anthocyanins, plant steroids, and flavonoids [112]. Their mode of action is based on altering cellular metabolic functions, stabilizing membranes, and defending photosynthetic and respiratory functions from ROS, synergistic acting with other enzymatic antioxidants and phytohormones. The AsA exerts a protective action against peroxide, superoxide, and hydroxide radicals and singlet oxygen. At the same time, α-tocopherol protects the cell membrane against lipid peroxidation. The GSH and its oxidized form glutathione disulfide (GSSG) are abundantly present in the cytosol, the nucleus, and mitochondria. GHS is a cofactor of several antioxidant enzymes (e.g., glutathione peroxidase and glutathione transferase), eliminates hydroxyl radicals and singlet oxygen, and contributes to the regeneration of vitamins C and E [113].

The role of antioxidant compounds in the plants’ adaptation to heat stress was studied in several plant species. Tomato and watermelon plants grown under high temperatures showed a higher accumulation of soluble phenols than observed in plants grown under optimal conditions [114]. The increased accumulation and reduced oxidation of phenols were probably due to the increased enzyme activity of phenylalanine ammonia-lyase (PAL) and a lower activity in high temperatures induced by polyphenol oxidase (PPO) and peroxidases (POX). Wahid et al. [112] reported that the accumulation of anthocyanins caused a decrease in the osmotic leaf potential to maximize the absorption and prevent water loss through transpiration, as well as acting as a UV screen. In a recent trial on zucchinis grown under anti-insect nets, thermal stress increased the contents of hydrophilic and lipophilic antioxidant activity, total phenols, and total ascorbic acid [27]. Camejo et al. [94] underlined the photoprotective activity of carotenoids such as xanthophyll and terpenoids such as tocopherol in the stabilization of thylakoid membranes. At the same time, zeaxanthin produced by the hydroxylation of β-carotene performed similar functions in Arabidopsis [115]. Enzymatic antioxidants are usually considered the most effective anti-ROS tools [116].

The first defense system of the plant is the SOD, which catalyzes the dismutation of the toxic superoxide anion O_2_^•−^ to molecular oxygen and H_2_O_2_:

$$2{\;O}_{2}^{•-\;}+2H^{+}\overset{\mathrm{SOD}}{\to }{\;H}_{2}O_{2}+O_{2}$$

The hydrogen peroxide produced will act as a substrate for CAT and APX. The CAT is an oxidoreductase of hydrogen peroxide and catalyzes the dismutation of H_2_O_2_ to water and oxygen:

$$2H_{2}O_{2}\overset{\mathrm{CAT}}{\to }\;2H_{2}O+O_{2}$$

However, the antioxidant compounds play a crucial role in activating the ascorbate-glutathione (AsA-GHS) cycle involved in ROS detoxification [76].

**Figure 1:**
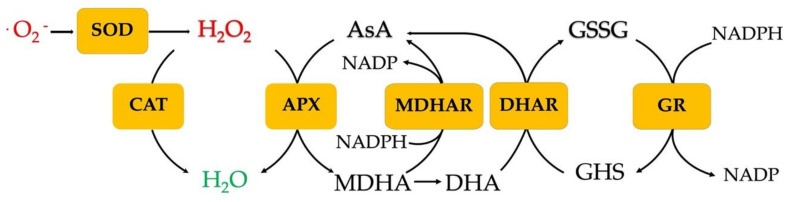
Enzymatic and nonenzymatic active antioxidants in plant defense and the Foyer-Halliwell-Asada cycle (also known as the AsA-GHS cycle) with its intermediates are reported. The Foyer-Halliwell-Asada cycle starts with the reduction of hydrogen peroxide in water by ascorbate peroxidase (APX). Abbreviations: SOD, superoxide dismutase; CAT, catalase; APX, ascorbate peroxidase; MDHAR, monodehydroascorbate reductase; DHAR, dehydroascorbate reductase; GR, glutathione reductase; MDHA, monodehydroascorbate reductase; DHA, dehydroascorbate reductase; GHS, reduced glutathione; and GSSG, glutathione disulphide.

The ascorbate-glutathione cycle (AsA-GHS) or Foyer-Halliwell-Asada pathway (Figure 1) includes a series of chemical cascade reactions, described below:

First, the APX catalyzes the reduction of H_2_O_2_ to H_2_O utilizing ascorbate as a specific electron donor:

$$2H_{2}O_{2}+\;\mathrm{AsA}\overset{\mathrm{APX}}{\to }\;2H_{2}O+2\mathrm{MDHA}$$

The monodehydroascorbate (MDHA) is regenerated by monodehydroascorbate reductase (MDHAR):

$$\mathrm{NADH}+{\;H}^{+}+2\mathrm{MDHA}\;\overset{\mathrm{MDHAR}}{↔}{\mathrm{NAD}}^{+}+2\mathrm{AsA}$$

However, monodehydroascorbate, if not rapidly reduced, breaks down into ascorbate and dehydroascorbate (DHA). Dehydroascorbate (DHA) is reduced to ascorbate and oxidized glutathione (GSSG) by dehydroascorbate reductase (DHAR):

$$2\mathrm{GSH}+\;\mathrm{DHA}\;\overset{\mathrm{DHAR}}{↔}\;\mathrm{GSSG}+\mathrm{AsA}$$

After eliminating the harmful hydroperoxide, the GSSG must return to its reduced form (GSH) to reacquire its antioxidant activity; this is achieved by an NADPH-dependent enzyme known as glutathione reductase (GR) through the following reaction:

$$\mathrm{GSSG}+\mathrm{NADPH}\;+{\;H}^{+}\overset{\mathrm{GR}}{\to }\;2\mathrm{GSSG}+{\mathrm{NADP}}^{+}$$

### 4.4. Heat Stress Impact on Product Quality

Thermal stress influences the morpho-physiological aspects of vegetables, thus undermining the quality and causing significant economic loss. However, recent studies have shown that plants under moderate heat stress can exhibit better-quality features [117]. In protected environments, thermal stress induces physiological alterations and affects vegetables’ appearance, flavor, carbohydrate content, and aromatic and antioxidant compounds. 

For example, if white asparagus is exposed to thermal stress, the rapid opening of the heads induces purple coloration, thus reducing their quality and economic value; moreover, an increase in fibrousness, wilting of shoot tips, and imbalances in calcium assimilation were also observed [118,119]. Studies on onions revealed an increase in sulfur compounds (important for flavor) as the temperature increased, as well as bulb splitting [119,120]. Similarly, carrot cultivars exposed to high temperatures showed a better and more intense taste and an increased terpenes content but a carotene reduction [121]. In broccoli, temperatures around 25 °C caused head deformation, premature ripening, and discoloration [122]. However, as reported by Mølmann et al. [123], high temperatures induced a higher accumulation of anthocyanins, glucosinolates, phenols, and flavonoids that led to a less sweeter taste than in broccoli that was exposed to lower temperatures (12 °C). Similar findings were obtained in Chinese cabbage [124]. In lettuce, temperatures above 15–18 °C determined a higher incidence of physiological disorders, such as loose head, tipburn, and leaf chlorosis. In contrast, a higher accumulation of bitter compounds and vitamins C and E but a lower accumulation of carotene were recorded [117,119,125,126]. Similarly, in tomatoes, heat stress led to an increase in vitamin C content and antioxidant compounds, contrasted by a decrease of the lycopene content and macronutrients such as magnesium, calcium, and potassium. Additionally, for peas, tomatoes, melons, and watermelons, a lower sugar content was observed [119,124,126].

Several studies showed a relationship between the expression of antioxidant enzymes, temperature, and genetic tolerance to heat stress. The scientific literature suggests explicitly that antioxidant activity increases over a range of certain temperature levels. Chakrabortty and Pradhan [127] reported that catalase, ascorbate peroxidase, and superoxide dismutase enzymes increased up to 50 °C. On the other hand, the activity of peroxidase and glutathione reductase demonstrated a decrease in the temperature range of 20–50 °C. 

Temperature is not the only variable to play an important role in enzymatic antioxidant activation and expression. Studies on field crops indicate that the expression of antioxidant enzymes increases in heat-resistant species at all stages of growth. For example, there was a higher accumulation of GHS and GHS/GSSG ratio [128], GST (glutathione S-transferase), POX, APX, CAT, SOD, and GR [129,130]. 

## 5. Conclusions

Scientists and producers are being motivated by climate change and consumers’ appreciation of healthy foods to broaden their vision on conventional production processes. In particular, this is encouraging them to adopt multidisciplinary approaches to improve productivity, including novel breeding targets, pest control strategies, and stress reduction tools. The introduction of insect-protection physical measures has provided a safe tool for the environment, offering the suitable defense against harmful insects, as well as new alien species, as part of the attempts to increase greening and environmental sustainability. Nowadays, growers have a wide range of insect nets available that differ in manufacturing and performance, helping them to choose the most suitable ones for their purposes. However, the use of anti-insect nets demands careful assessment of the effects they have on the microclimate, particularly in warm climatic regions, where the radiation surplus can cause a rapid and detrimental increase in temperature that will ultimately has to be overcome to avoid a significant drop in production or, in exceptional circumstances, the total loss of production. In a planet exposed to global warming, there is an urgent need to draw the attention of engineers, producers, and researchers to find the right compromise between insect protection and favorable climatic conditions for plant growth. Researchers have focused most of their attention on improving the airflow of anti-insect nets to avoid detrimental increases in temperature and suboptimal growth environments while continuing to exclude insects and not affecting the quality of the final product. Most of this research was conducted in a simulated environment using computational fluid dynamics (CFD) models. It is now necessary to increase knowledge on more realistic growth conditions and to study the insect net interaction with crops. The reviewed literature indicated that high temperatures induce high adaptive responses in edible vegetables. Plants’ defense mechanism of producing antioxidant compounds against harmful ROS is an excellent quality boost for vegetables until a certain threshold. Given these considerations, we believe that it is necessary to investigate these aspects to develop mathematical models that can predict the performance of insect nets in more realistic conditions to be able also to correlate it with vegetable qualities. These models would make it possible to develop versatile insect nets that can provide physical protection, improve airflow, and increase the quality of vegetables while preserving the yields.

## Figures and Tables

**Table 1:** Hypothetical exclusion efficiency ^1^ of insect nets for the control of a designated pest, hole size, and mesh number of widespread insect nets and average thorax width of “key insects”.

Insect Species	Screen Hole Size		Average Thorax Width ^4^ (μm)					
	Microns	Mesh	Male	Female	Male	Female	Male	Female
*Frankliniella occidentalis * ^2^	192	132	190.6	258.0	184.4	245.5	215	
*Bemisia argentifolii*	239	---	---	---	---	---	239	
*Trialeurodes vaporarium*	288	---	---	---	---	---	288	
*Aphis gossypii*	340	78	486.3		355		355	
*Bemisia tabaci*	462 ^3^	52	241.7	277.5	215.8	261.3	---	
*Myzus persicae*	---	---	---	---	433.8		---	
*Liriomyza trifolii*	640	40	---	---	562.5	653.8	608	
Reference	[46]		[35]		[32]		[46]	

^1^ An insect net is theoretically effective when the width of its pores is equal or less than the thorax width of the insect to be excluded. ^2^ Thrips (*Frankliniella occidentalis*) are very thin and can pass through common nets. ^3^ Thoracic width and hole size are not the only parameters to predict the efficacy of insect exclusion; hole geometry and the way in which holes were formed are crucial elements as well. ^4^ In this table, the thorax width was measured in the dorsal view.

**Table 2:** Evaluation of anti-insect screens with different discharge coefficients (C_d_), porosity (ε), and mesh sizes on the temperature differences (ΔT) and humidity between the inside and outside of the greenhouses under real conditions and with computational fluid dynamics (CFD) simulation models.

Experimental Conditions	Treatments	Effect on Microclimate	Reference
Simulation model	Evaluation of a model to predict the effect of screen area/opening area ratio on ΔT (inside/outside). Net radiation and wind velocity were set to 500 Wm^−2^ and 1 ms^−1^, respectively.	For a screen area/opening area ratio of one, the nets with a discharge coefficient of 0.1 and 0.5 resulted in a ΔT of 0.75 °C and 4.5 °C, respectively.	[58]
Multi-span greenhouse	Effect on inner temperature and humidity of two insect screens with different porosities (ε = 0.5 and ε = 0.6)	Anti-insect nets with porosity of 0.5 and 0.6 resulted in 2.5 and 2-fold increase in ΔT, respectively, compared to the unscreened greenhouse.	[50]
Four-span greenhouse	Effect on inner temperature and humidity of two insect screens with different porosities (ε = 0.2 and ε = 0.4) mounted on the roof and side openings of a four-span greenhouse.	Anti-insect nets with porosity of 0.2 and 0.4 resulted in 3 and 2-fold increases in air temperature and humidity, respectively, compared to the unscreened greenhouse.	[69]
Greenhouse	Effect of anti-thrips net (C_d_ = 0.22) on air temperature in a greenhouse in the tropical region with small plants and low transpiration rate.	Unripe plants (low transpiration rate) grown under the anti-thrips net led to a temperature increase of 5 °C. Differently, mature plants (high transpiration) under anti-thrips net showed a temperature of 3 °C.	[70]
Greenhouse	Effects of insect nets with different porosities (53%, 34%, 33%, and 19%) on vertical temperature distribution in greenhouses with tomato crops at two different growth stages and two densities.	Fine net porosity resulted in a higher air temperature. The highest temperature peak was recorded at the eaves height of the greenhouse. Taller plants and higher plant density resulted in lower air temperatures at all vertical points.	[71]
CFD simulation model	Evaluation of anti-Bemisia (ε = 0.41) and anti-thrips (ε = 0.2) nets positioned on the roof alone and roof and side openings of a multi-span greenhouse on the inner microclimate.	Both nets led to a significant increase in temperature, as compared to the unscreened control. Specifically, unscreened control, anti-Bemisia, and anti-thrips nets resulted in ΔT of 2.4 7.1, and 5.1 °C, respectively.	[72]
Greenhouse	Effects of different mesh sizes of nets (40, 52, and 78 mesh) on microclimate and air exchange rates in the humid tropics.	The 78 and 52-mesh nets increased air temperatures of 1–3 °C. In addition, the 78-mesh net determined an increase in humidity of about twice as much as observed with the 40-mesh net, while 52-mesh net led to a rise of 50%.	[73]
Mono-span greenhouse	Influence of different vent opening positions (side-only, roof-only, and combined roof and side openings) and anti-aphid insect screens on the microclimate.	The combined application of roof and side openings resulted in a reduction of the air temperature in the greenhouse compared to the roof or side vents alone.	[74]
